# Risk factors for the development of endometrial lesions in breast cancer patients using tamoxyphen: a retrospective cohort study

**DOI:** 10.1590/0100-6991e-20233442-en

**Published:** 2023-03-13

**Authors:** ALVO ORLANDO VIZZOTTO, SERGIO MANCINI NICOLAU, GUILHERME MUNHOZ LOPES, ADAUTO CASTELO

**Affiliations:** 1 - Hospital Santa Rita de Maringá, Serviço de Oncologia - Maringá - PR - Brasil; 2 - Universidade Federal de São Paulo - Escola Paulista de Medicina, Departamento de Ginecologia - São Paulo - SP - Brasil; 3 - Hospital Sírio Libanês, Instituto de Ensino e Pesquisa - IEP - São Paulo - SP - Brasil; 4 - Universidade Federal de São Paulo - Escola Paulista de Medicina, Departamento de Medicina - São Paulo - SP - Brasil

**Keywords:** Breast Neoplasms, Tamoxifen, Risk Factors, Uterine Diseases, Obesity, Neoplasias da Mama, Tamoxifeno, Fatores de Risco, Doenças Uterinas, Obesidade

## Abstract

**Introduction:**

breast cancer is the cancer with the highest incidence in women in Brazil, representing 29.7% of all cancers. More than two thirds of women with breast cancer show expression for hormone receptors, and in these cases, hormone therapy with tamoxifen is indicated, which may represent a risk factor for the development of endometrial cancer (four-fold greater relative risk).

**Objective:**

this study aimed to evaluate the association of tamoxifen and the development of endometrial disturbances and to assess possible other associated risk factors.

**Patients and method:**

a total of 364 breast cancer patients were evaluated, 286 who used tamoxifen and 78 who did not use this hormone therapy. Results: patients who used tamoxifen had a mean follow-up time of 51.42 months similar to those without hormone therapy (p=0.081). A total of 21 (7.3%) women who used tamofixen and no cases among women without hormone therapy presented endometrial changes during follow-up (p=0.01). Despite information regarding obesity was available for only 270 women, obesity was also significantly associated with the development of endometrial changes (p=0.008).

**Conclusion:**

furthermore, the association between tamofixen and endometrial changes remained significant (p=0.039) after adjusting for obesity.

## RESUMO

**Introdução:**

o câncer da mama é o câncer de maior incidência no sexo feminino no Brasil, representando 29,7% de todos os cânceres. Mais de dois terços das mulheres com câncer da mama apresentam expressão para receptores hormonais, estando, nestes casos, indicada a terapia hormonal com tamoxifeno, que pode representar fator de risco para o desenvolvimento do câncer do endométrio (risco relativo quatro vezes maior).

**Objetivo:**

este trabalho teve como objetivo avaliar a associação entre o uso de tamoxifeno e o desenvolvimento de distúrbios endometriais bem como eventuais outros fatores associados.

**Pacientes e método:**

Estudo de coorte retrospectivo de 364 pacientes com câncer da mama, das quais 286 utilizaram tamoxifeno e 78 não utilizaram esta hormonioterapia.

**Resultados:**

pacientes que usaram tamoxifeno tiveram um seguimento médio de 51,42 meses, semelhante àquelas sem terapia hormonal (p=0,081). Um total de 21 (7,3%) mulheres que usaram tamofixeno e nenhuma mulher sem terapia hormonal apresentaram alterações endometriais durante o seguimento (p=0,01). Nas 270 mulheres que tinham informação sobre obesidade, esta se associou significantemente com o desenvolvimento de alterações endometriais (p=0,008). A associação entre tamofixeno e alterações endometriais permaneceu significante (p=0,039) após ajustar para interação com obesidade.

**Conclusão:**

o uso de tamoxifeno no tratamento do câncer da mama esteve associado ao maior risco para desenvolvimento de alterações endometriais especialmente quando associado à obesidade.

## INTRODUCTION

Breast cancer is the malignancy with the highest incidence in females in Brazil, with 66,280 new cases estimated for 2020, representing 29.7% of all cancers according to data from the Brazilian National Cancer Institute (INCA)^1^. More than two-thirds of women with breast cancer have hormone receptors expression (positive estrogen receptor / positive progesterone receptor: ER+/PR+; positive estrogen receptor / negative progesterone receptor: ER+/PR-; negative estrogen receptor / positive progesterone receptor: ER-/PR+), and hormone therapy is often used in such cases, especially tamoxifen^2^.

Tamoxifen in the hormonal treatment of breast cancer is a risk factor for the development of endometrial diseases, especially endometrial cancer, with studies showing an increase in this risk by more than 4 times (Bernstein et al., 1999)^3^. Thus, in this group of patients, careful clinical follow-up would be recommended, associating imaging propaedeutics for endometrial evaluation.

Given the above, the aim of this study was to evaluate the development of endometrial disorders in women with breast cancer exposed to tamoxifen and assess which factors could be associated with it.

## PATIENTS AND METHODS

This study has a retrospective cohort design.

We evaluated women with breast cancer treated at the Oncology Service of a general hospital in the interior of Paraná, between 2010 and 2020, diagnosed with breast carcinoma and with a minimum follow-up of six months. We excluded patients with other types of breast tumors, as well as those whose records did not contain information on the start date of hormone therapy in the group that received tamoxifen or on the date of surgery in the group without hormone therapy.

We collected data retrospectively, through access to the electronic medical records, after approval by the Ethics and Ethics in Research Committees and registration on the Brazil Platform.

The patients were divided into two groups:

- Group 1 (G1): patients with a positive immunohistochemical reaction for hormone receptors (ER+/PR+, ER+/PR-, ER-/PR+), treated with tamoxifen.
- Group 2 (G2): patients with negative immunohistochemical reaction for hormone receptors (ER-/PR-) or who have not received hormone therapy with tamoxifen.

In both groups, we evaluated the development of endometrial disorders, such as endometrial thickening on ultrasound (thickness ≥10mm), endometrial hyperplasia, endometrial polyp, or endometrial cancer. In addition, we collected data on the associated factors obesity, arterial hypertension, and diabetes mellitus. 

We compared the frequency of associated factors in the two groups using the Chi-square test with Pearson’s correction, and the follow-up time for each group using the Student’s t test for independent samples. We also used the chi-square to analyze the association between exposure to tamoxifen, as well as the associated factors, and the onset of endometrial disorders. As the time of exposure to tamoxifen was not homogeneous, we assessed the risk of endometrial disease with survival curves (Kaplan-Meier) in both groups. We applied this same analysis to compare the occurrence of the outcome between obese and non-obese women. We performed the statistical analyzes with the SPSS^®^ version 21 software.

## RESULTS

We evaluated 466 medical records of female patients with breast cancer treated between 2010 and 2020. Three patients whose breast neoplasms were not carcinoma were excluded from the analysis. Of the remaining, 349 patients received hormone therapy. Of these, 310 received adjuvant tamoxifen at the standard daily dose of 20mg, 286 of them for more than six months; 78 women did not receive hormone therapy with tamoxifen. Thus, 364 patients were eligible for analysis: 286 who received tamoxifen for more than six months and 78 who did not receive hormone therapy with tamoxifen.

The mean age of 56.7 years, median of 57 years, and 69.76% were between 40 and 69 years old. 

The mean follow-up time was 51.42 months for patients who used tamoxifen and 59.73 months for those who did not receive this hormone therapy (p=0.081).

The clinical stage (FIGO 2021 and UICC 2018) of the patients at diagnosis is shown in Table 1. 

**Table 1:** Distribution of clinical staging.

CS	TNM	n
0	Tis, N0, M0	8
IA	T1, N0, M0	79
IB	T0, N1mi, M0; T1, N1mi M0	0
IIA	T0, N1, M0; T1, N1, M0; T2, N0, M0	119
IIB	T2, N1, M0; T3, N0, M0	80
IIIA	T0, N2, M0; T1, N2, M0; T2, N2, M0; T3, N1, M0; T3, N2, M0	21
IIIB	T4, N0, M0; T4, N1, M0; T4, N2, M0	28
IIIC	Any T (Tis, T1, T0, T2, T3, T4); N3, M0	6
IV	Any T (Tis, T1, T0, T2, T3, T4); Any N (N0, N1mi, N1, N2, N3); M1	23

CS: Clinical staging (FIGO 2021); TNM classification (UICC 2018); n: number of patients.

Regarding the tumor subtype according to the immunohistochemical findings, the patients were distributed as follows: 151 patients had luminal A (ER+/PR+ Ki67<15%); 111, luminal B (ER+/PR+ Ki67 ≥15%); 63 cases, hybrid luminal (ER+/PR+, HER2+++/ positive Fish); 20 individuals, HER2 subtype (ER-/PR-, HER2+++/ positive Fish) and 19 patients triple negative (ER-/PR-, HER2 0, +, ++/Fish negativo) (Table 2).

**Table 2:** Distribution according to tumor subtype (immunohistochemical findings):

LA - luminal A (ER+/PR+ Ki67<15%, HER2 0, +, ++/Fish negative)	151
LB - luminal B (ER+/PR+ Ki67 ≥15%, HER2 0, +, ++/Fish negative)	111
HL - hybrid luminal (ER+/PR+, HER2+++/Fish positive)	63
HER2 - (ER-/PR-, HER2+++/ Fish positive)	20
Triple negative - (ER-/PR-, HER2 0, +, ++/Fish negative)	19

ER: Estrogen Receptor; PR: progesterone receptor; HER2: presence of the her-2 gene expression product receptor.

Table 3 lists the frequency of risk factors in the two groups of women with and without tamoxifen.

**Table 3:** Frequency of risk factors in groups of women who used and did not use tamoxifen.

	Tamoxifen		
	Yes	No	p
Obesity	34	13	0,36
Systemic arterial hypertension	78	25	0,45
Diabetes mellitus	26	7	0,91

Of the 286 women treated with tamoxifen, 21 (7.3%) had some type of endometrial alteration, and eight had endometrial thickening (endovaginal pelvic ultrasound showing endometrial echo >10mm) and were not submitted to any further investigative procedure. The other 13 patients underwent uterine curettage, hysteroscopy, or hysterectomy, whose anatomopathological studies showed the presence of endometrial hyperplasia in eight cases, endometrial polyp in two, endometrial adenocarcinoma in one case, atrophic endometrium in one, and submucous leiomyoma in one case (Table 4).

**Table 4:** Endometrial changes.

Endometrial thickening	8
Endometrial hyperplasia	8
Endometrial polyp	2
Adenocarcinoma	1
Atrophic endometrium	1
Submucosal leiomyoma	1
Total	21

In the 78 patients who did not receive hormone therapy, there were no cases of endometrial disease.

**Figure 1:**
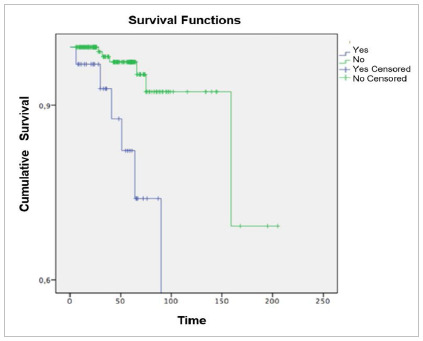
Evolution of the outcome (endometrial changes) in patients with and without obesity (time in months).

The use of tamoxifen was significantly associated with the development of endometrial changes, as shown in Table 5 and Figure1 (p=0.014).

**Table 5:** Hormone therapy and endometrial changes.

	Endometrial changes	No endometrial changes	Total
With hormone therapy	21	265	286
Without hormone therapy	0	78	78
Total	21	366	364

p=0.014 (Pearson chi-square).

Table 6 shows the univariate association of the assessed risk factors with the development of endometrial changes during follow-up. Only obesity showed a significant association with the development of endometrial changes during follow-up p=0.001.

**Table 6:** Association between risk factors and development of endometrial changes (outcome) in the group of women exposed to tamoxifen.

		Outcome		
		Yes	No	p
Obesity*	Yes	6 (17,6%)	28 (82,4%)	0,001
	No	6 (3,4%)	169 (96,6%)	
Systemic arterial hypertension	Yes	3 (3,8%)	75 (96,2%)	0,241
	No	13 (7,8%)	153 (92,2%)	
Diabetes mellitus	Yes	1 (3,8%)	25 96,2%)	0,548
	No	16 (6,9%)	226 (93,1%)	

*RR= 5.17.

**Figure 2:**
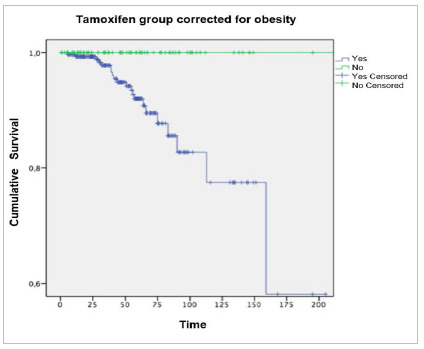
Outcome evolution (endometrial changes) in patients with and without tamoxifen use (time in months).

Figure 2 shows the association of obesity with the development of endometrial changes during follow-up according to their time of onset.

Regarding to arterial hypertension, among the women who used tamoxifen, 78 were hypertensive (27%); of those, three (3.8%) developed endometrial changes, versus 13 (7.8%) of the 166 patients without arterial hypertension (Table 6, p=0.241). Diabetes mellitus was also not associated with the outcome, with 3.8% and 6.9% in the groups exposed and not exposed to tamoxifen, respectively (p=0.548).

The assessment of the association of tamoxifen use adjusted for obesity is shown in Table 7 (Mantel-Haenzel chi-square, p=0.088).

**Table 7:** Relationship between tamoxifen use and endometrial changes adjusted for obesity.

				Outcome		
Obesidade				Yes	No	Total
Yes	Ht	Yes	Score	6	28	34
			% In ht	17.6%	82,4%	100,0%
		No	Score	0	13	13
			% In ht	0.0%	100,0%	100,0%
	Total		Score	6	41	47
			% In ht	12.8%	87,2%	100,0%
No	Ht	Yes	Score	6	169	175
			% In ht	3.4%	96,6%	100,0%
		No	Score	0	48	48
			% In ht	0.0%	100,0%	100,0%
	Total		Score	6	217	223
			% In ht	2.7%	97,3%	100,0%
Total	Ht	Yes	Score	12	197	209
			% In ht	5.7%	94,3%	100,0%
		No	Score	0	61	61
			% In ht	0.0%	100,0%	100,0%
	Total		Score	12	258	270
			% In ht	4.4%	95,6%	100,0%

p=0.088 (Mantel-Haenzel chi-square); HT: hormone therapy.

## DISCUSSION

In the present study, the incidence of endometrial disorders occurred in 7.3% of the patients who received this medication for more than six months and in none of those without hormone therapy. Although it was not possible to assess the risk (odds ratio) of hormone therapy in the development of endometrial changes, as in the present sample patients who did not receive hormone therapy with tamoxifen did not develop endometrial changes, the difference in the incidence of such changes between groups was statistically significant (p =0.014).

The use of tamoxifen as an adjuvant therapy for breast cancer is an effective and widely used treatment in these patients, as already demonstrated in several studies in the literature^3^ ^,^ ^4^ ^,^ ^14^ ^,^ ^18^. Although its effect on the endometrium is controversial^6^, its administration for long periods is related to the appearance of endometrial alterations, possibly due to an “estrogenic action” on the endometrium, although it is an antagonist of the receptor of this hormone^7^ ^-^ ^9^. The incidence of endometrial diseases in the group of women using tamoxifen for the treatment of breast cancer is quite variable in the literature, between 29% (Fisher et al., 2005)^4^ and 61% (Exacoustós et al., 1995)^5^. Bernstein et al. (1999)^3^ showed a risk 1.52 times greater for endometrial cancer in patients who used tamoxifen, reaching 4.06 times greater when this use lasted for more than five years. Our findings corroborate the data presented in the literature.

In several studies^8^ ^-^ ^10^, obesity was a risk factor significantly associated with the development of endometrial diseases, especially endometrial cancer, in women with breast cancer using tamoxifen. In this study, obesity was present in 16.3% of the women who used tamoxifen and in 21.3% of those who did not receive it. In the group that used tamoxifen, obesity increased the relative risk of developing endometrial disease by 5.17 when compared with non-obese women who took tamoxifen, this increase being significant (p=0.001). The assessment of the association of tamoxifen use adjusted for obesity was hampered by the lack of information on obesity in 77 patients who used tamoxifen, reducing the number of outcomes from 21 to 12, considerably decreasing the power of detecting the difference between exposed and non-exposed between obese and non-obese women (Table 7) (Mantel-Haenzel chi-square, p=0.088).

Arterial hypertension, present in 27% of women who used tamoxifen and in 29% of women who did not use it, was not associated with endometrial changes during follow-up (p=0.241).

Likewise, when analyzing the relationship between diabetes mellitus and the development of endometrial changes, we observed no difference between the exposed and non-exposed groups (p=0.548).

In view of the above, the present work allows us to conclude that the use of tamoxifen in the treatment of breast cancer was associated with a greater risk for the development of endometrial alterations, and the presence of obesity was significantly associated with a greater risk for the development of such alterations. Systemic arterial hypertension and diabetes mellitus were not associated with the development of endometrial changes. 
